# Cardiovascular mortality in patients with advanced chronic kidney disease with and without diabetes: a nationwide cohort study

**DOI:** 10.1186/s12933-023-01867-8

**Published:** 2023-06-16

**Authors:** Dea Haagensen Kofod, Nicholas Carlson, Ellen Freese Ballegaard, Thomas Peter Almdal, Christian Torp-Pedersen, Gunnar Gislason, Jesper Hastrup Svendsen, Bo Feldt-Rasmussen, Mads Hornum

**Affiliations:** 1 Department of Nephrology, Copenhagen University Hospital – Rigshospitalet, Inge Lehmanns vej 7, Copenhagen, 2100 Denmark; 2 Department of Endocrinology, Copenhagen University Hospital – Rigshospitalet, Copenhagen, Denmark; 3 Department of Clinical Medicine, Faculty of Health and Medical Sciences, University of Copenhagen, Copenhagen, Denmark; 4 Department of Cardiology, Copenhagen University Hospital – North Zealand, Hilleroed, Denmark; 5 Department of Public Health, University of Copenhagen, Copenhagen, Denmark; 6 Department of Cardiology, Copenhagen University Hospital – Herlev and Gentofte, Gentofte, Denmark; 7 Department of Cardiology, Copenhagen University Hospital – Rigshospitalet, Copenhagen, Denmark

**Keywords:** Chronic kidney disease, Cardiovascular mortality, Diabetes, Albuminuria, Cholesterol, Hemoglobin

## Abstract

**Background:**

Cardiovascular mortality and the impact of cardiac risk factors in advanced chronic kidney disease (CKD) remain poorly investigated. We examined the risk of cardiovascular mortality in patients with advanced CKD with and without diabetes as well as the impact of albuminuria, plasma hemoglobin, and plasma low-density lipoprotein (LDL) cholesterol levels.

**Methods:**

In a Danish nationwide registry-based cohort study, we identified persons aged ≥ 18 years with an estimated glomerular filtration rate < 30 mL/min/1.73m^2^ between 2002 and 2018. Patients with advanced CKD were age- and sex-matched with four individuals from the general Danish population. Cause-specific Cox regression models were used to estimate the 1-year risk of cardiovascular mortality standardized to the distribution of risk factors in the cohort.

**Results:**

We included 138,583 patients with advanced CKD of whom 32,698 had diabetes. The standardized 1-year risk of cardiovascular mortality was 9.8% (95% CI 9.6–10.0) and 7.4% (95% CI 7.3–7.5) for patients with and without diabetes, respectively, versus 3.1% (95% CI 3.1–3.1) in the matched cohort. 1-year cardiovascular mortality risks were 1.1- to 2.8-fold higher for patients with diabetes compared with those without diabetes across the range of advanced CKD stages and age groups. Albuminuria and anemia were associated with increased cardiovascular mortality risk regardless of diabetes status. LDL-cholesterol was inversely associated with cardiovascular mortality risk in patients without diabetes, while there was no clear association in patients with diabetes.

**Conclusions:**

Diabetes, albuminuria, and anemia remained important risk factors of cardiovascular mortality whereas our data suggest a limitation of LDL-cholesterol as a predictor of cardiovascular mortality in advanced CKD.

**Supplementary Information:**

The online version contains supplementary material available at 10.1186/s12933-023-01867-8.

## Background

Chronic kidney disease (CKD) is a major global health problem affecting an estimated 850 million people worldwide [1, 2]. Patients with CKD are at a high risk of cardiovascular disease and mortality [3, 4]. However, the underlying mechanisms for the strong association between CKD and cardiovascular outcomes remain poorly understood [4, 5]. Current international guidelines define CKD as abnormalities of kidney structure or function that are present for more than three months with implications for health [6]. Consequently, patients with CKD comprise a heterogenous group including those with preserved kidney function on one end and those with end-stage renal disease on the other end. Diabetes is the leading cause of CKD and a well-established risk factor of cardiovascular disease in the general population [7]. However, studies suggest that the impact of diabetes and other traditional cardiac risk factors such as hypertension and dyslipidemia attenuates with declining kidney function [8, 9]. Some studies even report an inverse relationship between traditional cardiac risk factors and cardiovascular mortality in patients with advanced CKD [10].

Prior studies investigating the association between CKD, diabetes and cardiovascular risk have predominantly reported on outcomes in early stage CKD [9, 11–14], and results from patients with an estimated glomerular filtration rate (eGFR) < 30 mL/min/1.73m^2^ not on dialysis remain limited. Furthermore, patients with eGFR < 25–30 mL/min/1.73m^2^ have largely been excluded from major cardiovascular trials. This also applies to more recent clinical trials examining cardiovascular treatment effects in patients with CKD, including the trials with sodium-glucose cotransporter-2 (SGLT2) inhibitors (canagliflozin in the CREDENCE trial [15]; dapagliflozin in the DAPA-CKD trial [16]), glucagon-like peptide-1 (GLP-1) receptor agonists (liraglutide in the LEADER trial [17]), and nonsteroidal mineralocorticoid receptor antagonists (finerenone in the FIDELIO-DKD and FIGARO-DKD trials [18, 19]).

Thus, data pertaining to the risk of cardiovascular mortality and the impact of key cardiac risk factors in patients with advanced CKD remains limited. Specific focus on eGFR < 30 mL/min/1.73m^2^ is needed to increase awareness and guide clinicians in identifying patients at high risk of death from cardiovascular causes. Based on multiple national registers containing comprehensive health care data, we examined the risk of cardiovascular mortality in patients with advanced CKD with and without diabetes and the impact of albuminuria, plasma hemoglobin, and plasma low-density lipoprotein (LDL) cholesterol.

## Methods

### Data sources

In Denmark, the healthcare system provides tax-funded public medical care for all residents, and comprehensive healthcare data is recorded in multiple national registers. Data from these registers can be cross-linked on an individual level through the unique personal identification number provided to every Danish citizen [20]. Laboratory data were extracted from the national Register of Laboratory Results comprising primary care, outpatient, and hospital results from four of five administrative regions using the Nomenclature, Properties and Units (NPU) coding system [21]. Data regarding redeemed prescription medication was retrieved from the Danish National Database of Reimbursed Prescriptions based on the Anatomical Therapeutic Chemical Classification System (ATC) codes [22, 23]. The Danish National Patient Register was used to identify comorbidities recorded via the 10th edition of the International Classification of Diseases (ICD-10) and the Nordic Medico-Statistical Committee Classification of Surgical Procedures (NCSP) [24, 25]. Finally, the cause of death was retrieved from the Danish Registry of Causes of Death based on ICD-10 codes and classified as cardiovascular (ICD-10 codes DI0-DI9) or non-cardiovascular mortality [24]. Identifying cardiovascular mortality through this approach has previously been employed in Danish studies [26, 27]. Supplementary Table S1 lists all administrative codes used in this study.

### Study design and population

We conducted a Danish registry-based retrospective cohort study identifying persons aged ≥ 18 years with an eGFR < 30 mL/min/1.73m^2^ from 1 January 2002 until 31 December 2018. The assessment of eGFR was based on recorded plasma creatinine using the CKD-EPI creatinine equation [28]. People with prior renal transplantation or immigrating < 5 years prior to study inclusion were excluded. Furthermore, to account for persons with kidney failure secondary to imminent death—and because we aimed to examine the long-term cardiovascular effects of advanced CKD—we excluded persons having a major cardiovascular event (myocardial infarction or stroke) or dying < 30 days after inclusion. Thus, the index date for analyses was defined as 30 days after the first registered eGFR < 30 mL/min/1.73m^2^. As reference group, every patient with advanced CKD was matched (exposure density matching) with four living individuals with an eGFR ≥ 30 mL/min/1.73m^2^ from the general Danish population on birth year and sex.

### Study exposures and covariates

Patients with concomitant diabetes were identified as persons having claimed ≥ 1 prescription of glucose-lowering drug within five years prior to index. The duration of diabetes was defined as the time from first prescribed glucose-lowering drug until index. Using prescribed glucose-lowering medication to identify diabetes has been previously validated with a positive predicative value of 96.6% [29].

Existence of comorbidities was based on diagnosis and procedure codes recorded within five years prior to index except hypertension that was defined as having prescribed ≥ 2 anti-hypertensive drugs within five years prior to index. Chronic dialysis treatment was based on the procedure code of chronic dialysis within three months prior to index. Concomitant medications were identified from prescribed medications within six months prior to index. Laboratory workup used the most recent recorded sample within one year prior to index. Albuminuria status was classified according to 24-hour urine collection or urinary albumin-to-creatinine ratio (UACR) as normoalbuminuria (< 30 mg/24-h or UACR < 30 mg/g), microalbuminuria (30–299 mg/24-h or UACR 30–299 mg/g), or macroalbuminuria (≥ 300 mg/24-h or UACR ≥ 300 mg/g).

### Study outcome and follow up

The outcome was cardiovascular mortality with non-cardiovascular mortality as competing risk. Patients were followed from the index day until death, emigration, or 31 December 2018, whichever occurred first.

### Statistical analyses

Baseline characteristics were presented as counts with percentage for categorical data. Mean with standard deviation (SD) was used for normally distributed data and median with interquartile range (IQR) for non-normally distributed data.

Unadjusted cumulative risk of cardiovascular, non-cardiovascular, and all-cause mortality was computed based on the Aalen-Johansen estimator. Multiple Cox regressions stratified for matching variables (age and sex) and adjusted for cardiovascular disease (heart failure, myocardial infarction, and stroke) were performed to calculate the hazard ratios (HR) for cardiovascular, non-cardiovascular, and all-cause mortality, respectively. Based on the cause-specific Cox regressions, the 1-year risk of cardiovascular, non-cardiovascular, and all-cause mortality, respectively, was calculated standardized to the distribution of risk factors of all patients in the sample [30]. The standardized risks represent a weighted average of the crude risks adjusted for differences between the populations with regards to age, sex, and cardiovascular disease. We subsequently performed subgroup analyses stratified by age group (18–49 years, 50–59 years, 60–69 years, 70–79 years, 80 years or older), sex, cardiovascular disease, and CKD stage (CKD stage 4: eGFR 15–29 mL/min/1.73m^2^; CKD stage 5: eGFR < 15 mL/min/1.73m^2^; CKD stage 5D: receiving dialysis). Standardized 1-year risks are reported with 95% bootstrap confidence interval (CI).

To further investigate risk factors of cardiovascular mortality, we calculated the standardized 1-year risk of cardiovascular mortality in models adjusted for age, sex, cardiovascular disease, and CKD stage stratified by baseline albuminuria status, plasma LDL-cholesterol level and plasma hemoglobin level, respectively. These analyses only included patients with a valid baseline sample, were performed separately in patients with and without diabetes, and further in subgroup analyses stratified by CKD stage. Moreover, the association between LDL-cholesterol and cardiovascular mortality was re-analyzed in sub-analyses including only patients without concomitant medication for hyperlipidemia.

All data management and analyses were performed in SAS (version 9.4; SAS Institute, Cary, NC, USA) and R (version 4.0.1; R Core Team (2019)). A P-value below 0.05 was considered statistically significant, and all statistical tests were two-tailed.

### Sensitivity analyses

To assess for possible period effect, the main results were re-analyzed stratified according to index year (year 2002–2008, 2009–2013, 2014–2018). Regarding possible risk of misclassification of acute kidney injury as CKD, the main results were re-analyzed in sensitivity analyses including only patients with two measured eGFR < 30 mL/min/1.73m^2^ ≥ 90 days apart and in analyses including only patients with two measured eGFR < 30 mL/min/1.73m^2^ ≥ 90 days apart with < 20% variation between first and second eGFR.

### Ethics

Danish register-based studies do not require formal ethics permission. The Danish Data Protection Agency has approved the use of the study data (ref. P-2019-191). Data were managed and analyzed within a secure research platform administered through Statistics Denmark where data and codes also are accessible for review on request. Full data sharing is not possible due to the risk of potential identification of persons.

## Results

**Fig. 1:**
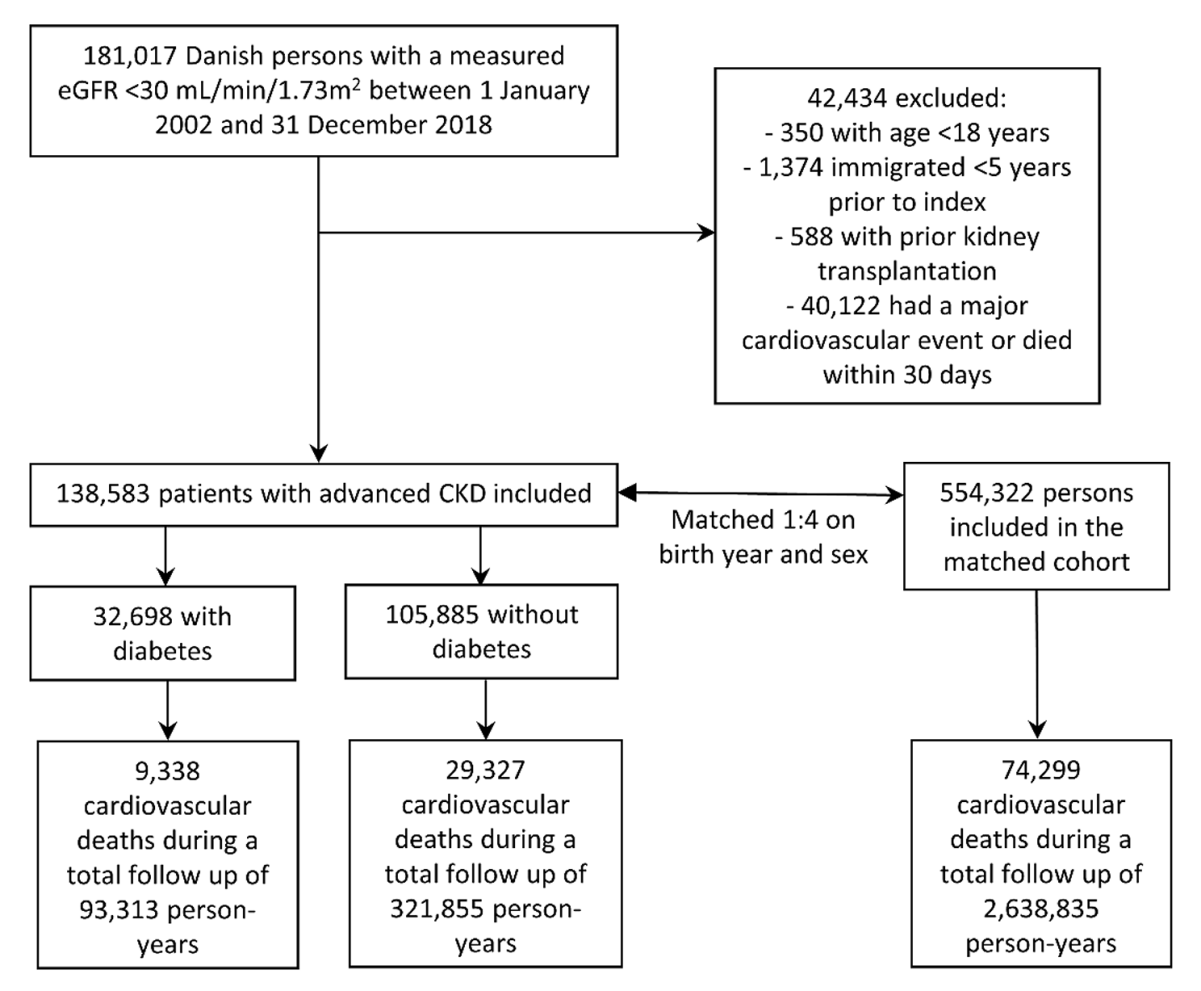
Flow of patients and the matched cohort. Flow diagram of identification and inclusion of patients with advanced chronic kidney disease (CKD) and the matched cohort.

Between 1 January 2002 and 31 December 2018, a total of 138,583 patients with advanced CKD were included of whom 32,698 were identified as having diabetes (Fig. 1). 554,322 individuals were included in the matched cohort. The median follow up was 2.1 (IQR 0.9-4.0) years for the diabetes group, 2.0 (IQR 0.6–4.2) years for the no-diabetes group, and 3.5 (IQR 1.7–7.1) years for the matched cohort. In total, there were 235,548 deaths during follow up of which 112,964 were cardiovascular with the following distribution between groups: 9,338 cardiovascular deaths out of 17,086 deaths for the diabetes group; 29,327 cardiovascular deaths out of 61,329 deaths for the no-diabetes group; and 74,299 cardiovascular deaths out of 157,133 deaths for the matched cohort.

### Baseline characteristics

Baseline characteristics of patients with advanced CKD with and without diabetes and the matched cohort are presented in Table 1. The median age was 75.4 (IQR 68.0-82.3) years, and 53.5% were men in the diabetes group. For patients without diabetes, the median age was 79.4 (IQR 70.2–86.3) years with a gender distribution of 46.4% men. The distribution across CKD stages were similar between the two groups with the majority identified as having CKD stage 4. The diabetes group had a significantly higher prevalence of all reported comorbidities compared with the no-diabetes group. For the matched cohort, the median age was 78.3 (IQR 69.5–85.4) years, 48.1% were men, 8.5% had diabetes, 11.2% had an eGFR < 60 mL/min/1.73m^2^, and 1.9% had albuminuria. Table 2 reports baseline diabetes characteristics for the diabetes group. The mean duration of diabetes was 9.7 (SD 6.0) years, and 35.8% received insulin treatment. The mean hemoglobin A1c level was 55.6 (SD 15.9) mmol/mol.

**Table 1:** Baseline characteristics of patients with advanced chronic kidney disease and the matched cohort

Characteristic	Matched cohort (n = 554,322)	No diabetes (n = 105,885)	Diabetes (n = 32,698)
Sex, n (%)			
Women	359,487 (51.9)	56,704 (53.6)	15,194 (46.5)
Men	333,418 (48.1)	49,181 (46.4)	17,504 (53.5)
Age [years], median (IQR)	78.3 (69.5–85.4)	79.4 (70.2–86.3)	75.4 (68.0-82.3)
Age distribution, n (%)			
18–49 years	23,776 (4.3)	4946 (4.7)	1042 (3.2)
50–59 years	34,758 (6.3)	6442 (6.1)	2318 (7.1)
60–69 years	86,620 (15.6)	14,916 (14.1)	6831 (20.9)
70–79 years	163,485 (29.5)	29,268 (27.6)	11,722 (35.8)
≥80 years	245,683 (44.3)	50,313 (47.5)	10,785 (33.0)
eGFR [mL/min/1.73m^2^], median (IQR)	72.6 (58.7–84.6)	25.8 (20.9–28.4)	26.2 (21.4–28.5)
Stage of CKD, n (%)			
CKD stage 4	-	93,535 (88.3)	29,212 (89.3)
CKD stage 5	-	11,156 (10.5)	3033 (9.3)
CKD stage 5D	-	1194 (1.1)	453 (1.4)
Comorbidities, n (%)			
Heart failure	19,231 (3.5)	15,962 (15.1)	6606 (20.2)
Previous myocardial infarction	11,706 (2.1)	6032 (5.7)	2438 (7.5)
Atrial fibrillation/flutter	40,231 (7.3)	18,888 (17.8)	6596 (20.2)
Previous stroke	19,879 (3.6)	7700 (7.3)	2619 (8.0)
Peripheral artery disease	8682 (1.6)	3908 (3.7)	2185 (6.7)
Lower extremity amputation	1458 (0.3)	489 (0.5)	873 (2.7)
Hypertension	195,168 (35.2)	66,523 (62.8)	27,187 (83.1)
Concomitant medication, n (%)			
Renin-angiotensin inhibitors	154,715 (27.9)	52,826 (49.9)	24,187 (74.0)
Diuretics	130,897 (23.6)	44,344 (41.9)	16,142 (49.4)
Acetylsalicylic acid	113,013 (20.4)	31,344 (29.6)	13,945 (42.6)
Lipid modifiers	122,223 (22.0)	30,360 (28.7)	20,991 (64.2)
Albuminuria status, n (%)			
Normoalbuminuria	21,648 (66.9)	4582 (33.1)	4051 (35.2)
Microalbuminuria	8675 (26.8)	4778 (34.5)	4302 (37.4)
Macroalbuminuria	2052 (6.3)	4484 (32.4)	3154 (27.4)
Plasma hemoglobin [mmol/L], mean (SD)	8.4 (1.0)	7.1 (1.2)	7.1 (1.1)
Total plasma cholesterol [mmol/L], mean (SD)	4.8 (1.1)	4.6 (1.3)	4.1 (1.2)
Plasma LDL-cholesterol [mmol/L], mean (SD)	2.6 (1.0)	2.5 (1.1)	2.0 (2.2)
Plasma HDL-cholesterol [mmol/L], mean (SD)	1.6 (0.5)	1.4 (0.5)	1.2 (0.4)

CKD denotes chronic kidney disease, eGFR estimated glomerular filtration rate, LDL low-density lipoprotein, and HDL high-density lipoprotein. Missing observations: eGFR n = 464,559 (matched cohort); albuminuria status: n = 521,947 (matched cohort), n = 92,041 (no diabetes), n = 21,191 (diabetes); hemoglobin: n = 455,285 (matched cohort), n = 35,922 (no diabetes), n = 8425 (diabetes); total cholesterol: n = 471,050 (matched cohort), n = 74,221 (no diabetes), n = 16,986 (diabetes); LDL-cholesterol: n = 481,546 (matched cohort), n = 70,407 (no diabetes), n = 15,362 (diabetes); HDL-cholesterol: n = 472,761 (matched cohort), n = 75,319 (no diabetes), n = 17,263 (diabetes)

**Table 2:** Baseline diabetes characteristics of patients with advanced chronic kidney disease and diabetes

Characteristic	Diabetes (n = 32,698)
Insulin treatment, n (%)	11,693 (35.8)
Non-insulin therapy, n (%)	23,088 (70.6)
GLP-1 receptor agonists, n (%)	1739 (5.3)
SGLT2 inhibitors, n (%)	391 (1.2)
Duration of diabetes [years], mean (SD)	9.7 (6.0)
Diabetic eye disease, n (%)	6082 (18.6)
Hemoglobin A1c [mmol/mol], mean (SD)	55.6 (15.9)

GLP-1 denotes glucagon-like peptide-1, and SGLT2 sodium-glucose co-transporter-2. Missing observations: Hemoglobin A1c n = 11,344

### Risk of cardiovascular mortality

**Fig. 2:**
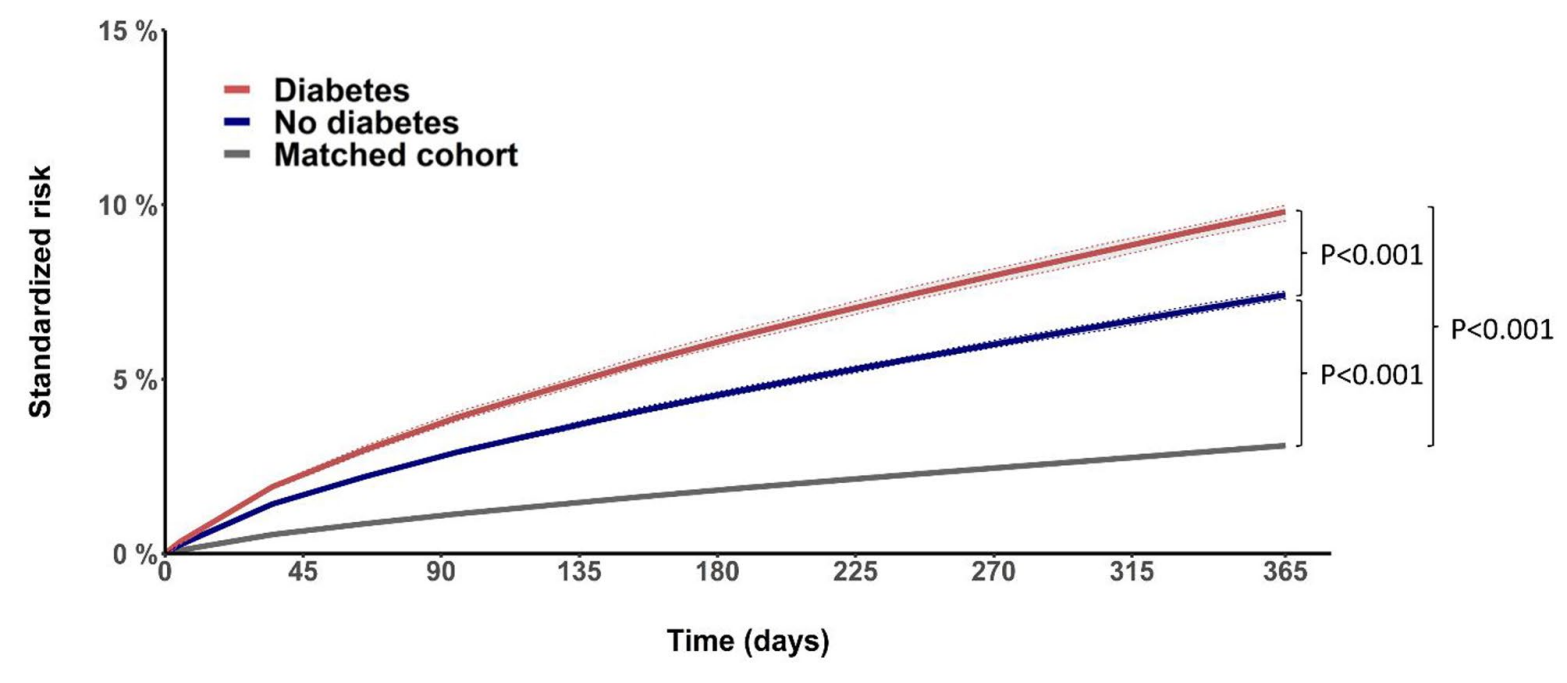
Cardiovascular mortality in patients with advanced chronic kidney disease and the matched cohort. Standardized risk with 95% CIs of cardiovascular mortality for patients with advanced chronic kidney disease, with and without diabetes, compared with an age- and sex-matched general population cohort

The standardized 1-year risk of cardiovascular mortality was dependent on diabetes (P for interaction < 0.001). The 1-year risk of cardiovascular mortality was 9.8% (95% CI 9.6–10.0) and 7.4% (95% CI 7.3–7.5) for patients with and without diabetes, respectively, versus 3.1% (95% CI 3.1–3.1) in the matched cohort (Fig. 2). The HR was 3.6 (95% CI 3.5–3.7, P < 0.001) for the diabetes group and 2.6 (95% CI 2.6–2.7, P < 0.001) for the no-diabetes group using the matched cohort as a reference. The unadjusted cumulative incidence of cardiovascular, non-cardiovascular, and all-cause mortality within one year is shown in supplementary Figure S1, and the standardized risk of non-cardiovascular and all-cause mortality is shown in supplementary Figure S2.

The HR and the 1-year risk of cardiovascular mortality stratified by age are presented in Table 3. Across all age strata, the risk of cardiovascular mortality was significantly higher for patients with advanced CKD compared with the matched cohort. Furthermore, patients with diabetes had a significantly higher risk of cardiovascular mortality compared with those without diabetes across all age strata although the risk ratio (RR) declined with increasing age: RR 2.8 (95% CI 2.3–3.3, P < 0.001) for age 18–49 years, RR 1.9 (95% CI 1.7–2.1, P < 0.001) for age 50–59 years, RR 1.4 (95% CI 1.4–1.5, P < 0.001) for age 60–69 years, RR 1.2 (95% CI 1.1–1.2, P < 0.001) for age 70–79 years, and RR 1.1 (95% CI 1.0-1.1, P < 0.001) for age ≥ 80 years. The analyses stratified by sex and prior cardiovascular disease are shown in supplementary Table S2. The risk of cardiovascular mortality was significantly higher for patients with advanced CKD compared with the matched cohort across all strata with the highest risk among patients with diabetes. Of note, prior cardiovascular disease markedly increased the risk for both patients with and without diabetes.

**Table 3:** Hazard ratios and standardized 1-year risk of cardiovascular mortality stratified by age

	Hazard ratio (95% CI)	Standardized 1-year risk (95% CI)	Standardized risk difference (95% CI)	Standardized risk ratio (95% CI)	P-value
**18–49 years**					
Matched cohort	Reference	0.07% (0.06–0.09)	Reference	Reference	
No diabetes	15.1 (12.1–19.0)	1.1% (1.0-1.2)	1.0% (0.9–1.2)	14.8 (11.6–19.0)	< 0.001
Diabetes	43.6 (34.1–55.8)	3.1% (2.7–3.6)	3.0% (2.6–3.5)	41.9 (33.0-54.6)	< 0.001
**50–59 years**					
Matched cohort	Reference	0.3% (0.2–0.3)	Reference	Reference	
No diabetes	9.4 (8.4–10.5)	2.3% (2.1–2.5)	2.0% (1.9–2.2)	9.0 (8.0–10.0)	< 0.001
Diabetes	17.8 (15.7–20.1)	4.3% (3.9–4.7)	4.1% (3.7–4.4)	16.8 (14.8–19.1)	< 0.001
**60–69 years**					
Matched cohort	Reference	0.7% (0.7–0.8)	Reference	Reference	
No diabetes	5.1 (4.8–5.4)	3.5% (3.4–3.6)	2.8% (2.6–2.9)	4.8 (4.6–5.1)	< 0.001
Diabetes	7.4 (7.0-7.8)	5.1% (4.8–5.3)	4.4% (4.1–4.6)	7.0 (6.5–7.4)	< 0.001
**70–79 years**					
Matched cohort	Reference	2.2% (2.2–2.2)	Reference	Reference	
No diabetes	2.8 (2.8–2.9)	5.9% (5.7-6.0)	3.7% (3.5–3.8)	2.7 (2.6–2.7)	< 0.001
Diabetes	3.3 (3.2–3.5)	6.9% (6.6–7.1)	4.7% (4.4–4.9)	3.1 (3.0-3.2)	< 0.001
**≥ 80 years**					
Matched cohort	Reference	5.2% (5.2–5.3)	Reference	Reference	
No diabetes	2.4 (2.3–2.4)	11.3% (11.1–11.5)	6.0% (5.9–6.2)	2.2 (2.1–2.2)	< 0.001
Diabetes	2.5 (2.4–2.6)	11.9% (11.4–12.3)	6.6% (6.2-7.0)	2.3 (2.2–2.3)	< 0.001

Table 4 reports the 1-year risk of cardiovascular and all-cause mortality across strata of CKD stages. Progressing CKD was associated with increased risk of mortality with dialysis treatment markedly augmenting the risk—especially for the diabetes group. Compared with the matched cohort, HRs of cardiovascular mortality at CKD stage 4, 5, and 5D were 3.5 (95% CI 3.4–3.6, P < 0.001), 4.0 (95% CI 3.7–4.3, P < 0.001), and 8.5 (95% CI 7.4–9.8, P < 0.001) for patients with diabetes, and 2.6 (95% CI 2.6–2.6, P < 0.001), 3.0 (95% CI 2.8–3.1, P < 0.001), and 5.6 (95% CI 5.1–6.1, P < 0.001) for patients without diabetes. The risk of cardiovascular mortality was significantly higher in patients with diabetes across all CKD stages compared with patients without diabetes.

**Table 4:** Standardized 1-year risk of mortality stratified by chronic kidney disease (CKD) stage

		No diabetes	Diabetes			
		Standardized 1-year risk (95% CI)	Standardized 1-year risk (95% CI)	Standardized risk difference (95% CI)	Standardized risk ratio (95% CI)	P-value
**Cardiovascular mortality**						
CKD stage 4		7.3% (7.2–7.4)	9.6% (9.4–9.8)	2.3% (2.1–2.5)	1.3 (1.3–1.4)	< 0.001
CKD stage 5		8.0% (7.6–8.3)	10.6% (9.8–11.4)	2.6% (1.7–3.4)	1.3 (1.2–1.4)	< 0.001
CKD stage 5D		14.1% (12.7–15.5)	19.5% (17.1–22.4)	5.4% (2.4–8.7)	1.4 (1.2–1.7)	< 0.001
**All-cause mortality**						
CKD stage 4		17.0% (16.9–17.2)	19.6% (19.3–19.9)	2.6% (2.2–2.9)	1.1 (1.1–1.2)	< 0.001
CKD stage 5		22.2% (21.5–22.8)	23.6% (22.4–24.7)	1.4% (0.0-2.8)	1.1 (1.0-1.1)	0.05
CKD stage 5D		27.5% (25.9–29.4)	36.4% (33.1–39.8)	8.9% (5.3–12.4)	1.3 (1.2–1.5)	< 0.001

### Risk factors of cardiovascular mortality

**Fig. 3:**
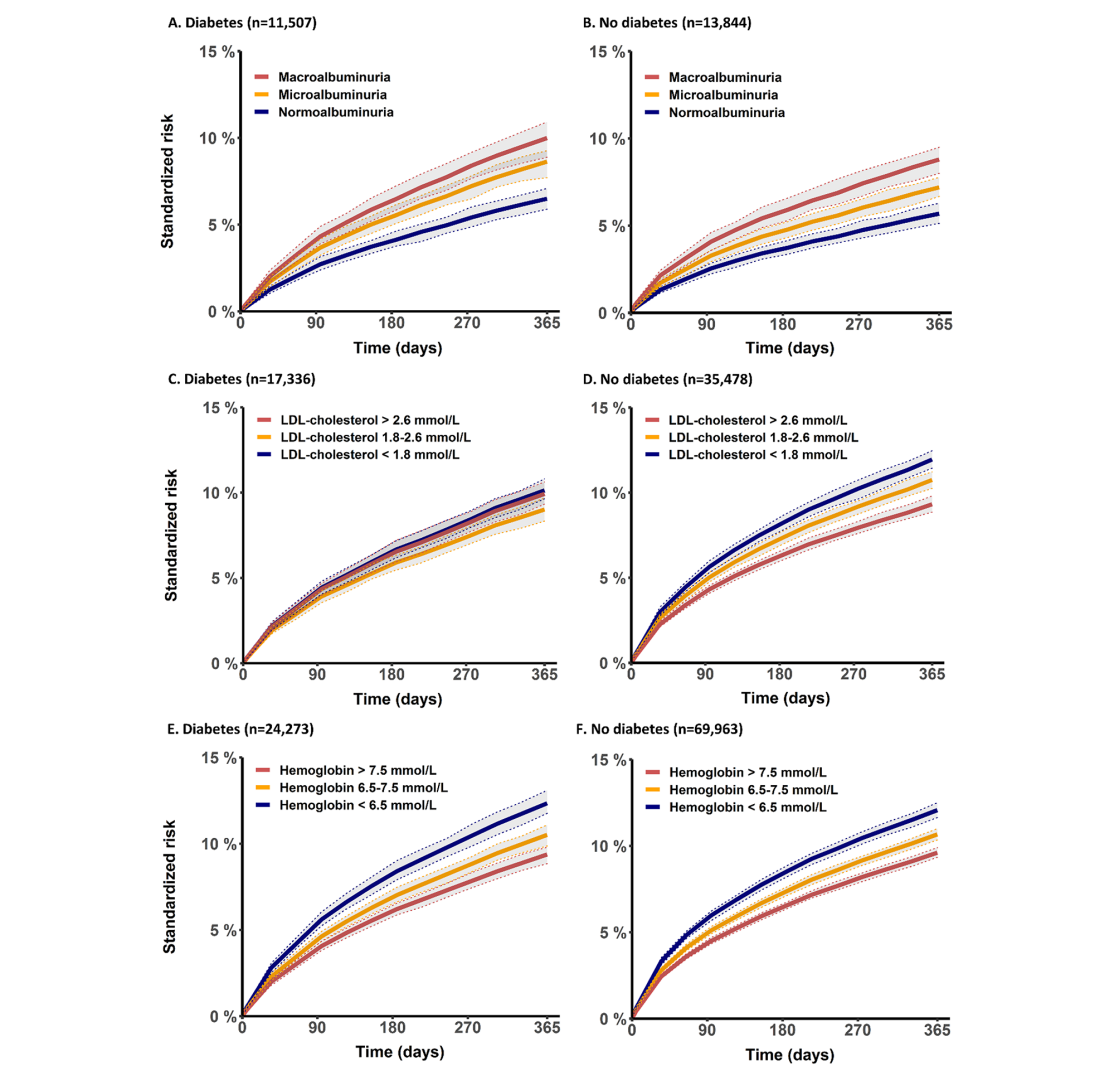
Cardiovascular mortality stratified by baseline laboratory measurements. Standardized risk with 95% CIs of cardiovascular mortality stratified by baseline albuminuria status (Panel A-B), plasma low-density lipoprotein (LDL) cholesterol level (Panel C-D), and plasma hemoglobin level (Panel E-F)

Figure 3 illustrates the association between the risk of cardiovascular mortality and albuminuria status, LDL-cholesterol level, and hemoglobin level, respectively. Albuminuria was associated with an increased risk of cardiovascular mortality for both patients with and without diabetes. We found no clear association between LDL-cholesterol level and cardiovascular mortality in the diabetes group. In patients without diabetes, we found an inverse relationship where an LDL-cholesterol level < 1.8 mmol/L was associated with an increased risk of cardiovascular mortality while an LDL-cholesterol level > 2.6 mmol/L was associated with the lowest risk. Of note, the same pattern was observed in the sub-analyses of only patients without concomitant medication for hyperlipidemia (supplementary Figure S3). Lastly, we found that a higher hemoglobin level was associated with a decreased risk of cardiovascular mortality. Overall, the effect of albuminuria, LDL-cholesterol, and hemoglobin level on cardiovascular mortality remained the same across CKD stages (supplementary Table S3).

### Sensitivity analyses

In the sensitivity analysis stratified by index year, the main results remained unchanged with a trend toward a decline in 1-year risk of cardiovascular mortality for all groups over time (supplementary Table S4). Furthermore, the main results remained unchanged in the analyses of patients with two measured eGFR values < 30 mL/min/1.73m^2^ ≥ 90 days apart, although the 1-year risk estimates were 2–3% higher in these cohorts (supplementary Tables S5 and S6).

## Discussion

In this large nationwide cohort, we found that patients with advanced CKD, with and without diabetes, had increased risk of cardiovascular mortality compared with an age- and sex-matched general population cohort. The cardiovascular mortality risk was highest in patients with concomitant diabetes, with a particularly pronounced risk amplification in young patients < 50 years of age where the relative risk was 41.9 compared with the matched cohort and 2.8 compared with those without diabetes.

While the association between CKD and cardiovascular mortality has been well-established, data regarding the risk of cardiovascular mortality specifically in people with eGFR < 30 mL/min/1.73m^2^ remain limited. Previous studies assessing the association between CKD and cardiovascular events have included a limited number of patients with CKD stage 4 and 5 and predominantly assessed patients with and without diabetes together [11–14, 31]. Based on data from 138,583 patients with eGFR < 30 mL/min/1.73m^2^, the present study estimated the standardized 1-year risk of cardiovascular mortality accounting for the competing risk of death from causes other than cardiovascular disease. With this method we provide risk estimates across the range of advanced CKD stages and various other subgroups separately in patients with and without diabetes.

Data regarding the impact of diabetes on cardiovascular risk in patients with advanced CKD is sparse and divergent. Fox et al. performed one of the few previous studies examining the risk of mortality throughout the ranges of eGFR in patients with and without diabetes separately [32]. They found that patients with diabetes had a higher rate of cardiovascular and all-cause mortality versus those without diabetes across the range of eGFR, but the effect of diabetes attenuated as kidney function declined. Two smaller studies found no association between diabetes and all-cause mortality in patients with advanced CKD; however, these studies may have been underpowered [33, 34]. Our data suggest that diabetes continues to be an important risk factor for cardiovascular mortality even in patients with advanced CKD. The effect was particularly evident in the younger age groups. To our knowledge, this is the first study to investigate the impact of diabetes on cardiovascular mortality in patients with advanced CKD in different age groups. Our findings add to existing knowledge by showing the importance of diabetes across the range of advanced CKD stages and age groups.

The impact of other traditional cardiac risk factors is also poorly understood in patients with advanced CKD. Some studies suggest that non-traditional uremia-related risk factors such as albuminuria and inflammation are increasingly important in predicting cardiovascular outcomes when kidney function declines [8, 10, 35]. In a systematic review and meta-analysis, Major et al. identified both traditional and non-traditional risk factors of cardiovascular events in non-dialysis dependent CKD. They found that diabetes, increasing age, and decreasing hemoglobin were associated with an increased rate of cardiovascular events, while lipid measurements, including LDL-cholesterol, did not have a clear relationship [36]. These results are consistent with our findings except that we found an inverse relationship between LDL-cholesterol and cardiovascular mortality in patients without diabetes. A previous study similarly reported an inverse relationship between LDL-cholesterol and cardiovascular mortality in men with moderate and advanced CKD [37]. The same pattern has been observed in patients receiving dialysis [38]. It has been suggested that the inverse relationship is caused by confounding by inflammation and malnutrition in those with low cholesterol levels [37, 38].

Our findings suggest that cardiovascular mortality is increased in patients with advanced CKD, both in the presence and absence of diabetes. The risk is particularly increased in patients with prior cardiovascular disease. Nevertheless, cardiovascular disease is frequently underdiagnosed and undertreated in advanced CKD [35]. Increased awareness of cardiovascular risk assessment in this high-risk population is important. Risk assessment might be improved by assessing uremia-related risk factors. Consistent with previous studies, we found that albuminuria was associated with an increased risk of cardiovascular mortality regardless of diabetes status [31, 32]. On the contrary, our data, together with previous studies, suggest a potential limitation of LDL-cholesterol as a predictor of cardiovascular mortality in patients with advanced CKD [36–38].

Optimal strategies for the prevention and treatment of cardiovascular disease in advanced CKD have yet to be determined. A recent meta-analysis examined the effectiveness of statin treatment in advanced CKD. The results indicated that statin treatment reduced major cardiovascular events in CKD stage 4, but too few patients with CKD stage 4 were included in lipid-lowering trials to draw confident conclusions. There was no effect of statin treatment in CKD stage 5/5D [39]. For patients with diabetes, improved glycemic control might improve outcomes, but no clinical trial has yet evaluated the effect of glycemic control on cardiovascular outcomes in advanced CKD [4]. Patients with advanced CKD have commonly been excluded from major cardiovascular trials, and extrapolation of cardiovascular prevention results in people with earlier stages of CKD or other populations might not be straightforward as the risk profile changes when kidney function declines. Clinical trials specifically in patients with advanced CKD are needed to improve the prognosis of this high-risk population.

There are some limitations to this study that are inherent in registry-based studies. The observational study design precludes causal inference. Unmeasured confounding cannot be excluded, and we were not able to obtain information regarding lifestyle, body mass index, or race. To define advanced CKD, we used a single measurement of eGFR which might have led to misclassification. However, principal results remained unchanged in sensitivity analyses based on two measured eGFR, and comparative analyses based on data in the Danish health care registers have previously reported no differences in 1-year risk of end-stage kidney disease and 1-year risk of mortality when comparing CKD cohorts using varying algorithms including one versus two eGFR measurements to identify patients with CKD [40]. Furthermore, misclassification of the cause of mortality cannot be excluded. The cause of death in registries is based on death certificates from available clinical information, and we note that the autopsy rate in Denmark is currently below 10% [41]. The proportion of patients with diabetes in our advanced CKD cohort corresponds to the proportions observed in previous studies in European countries [42, 43]. Baseline laboratory measurements were not available for all patients, and risk factor analyses based on baseline albuminuria status, LDL-cholesterol level, and hemoglobin level were only performed in the patients with advanced CKD. These analyses were conducted as complete case analysis and may be associated with detection bias. As a reference group, we used an age- and sex-matched cohort with an eGFR ≥ 30 mL/min/1.73m^2^. Consequently, some individuals in the matched cohort suffered from earlier stages of CKD whereby our risk estimates are relatively conservative. However, previous data suggest that GFR decreases as part of normal aging [44]. Because our advanced CKD cohort had a median age of 78 years, matching individuals with a higher eGFR (e.g. >60 mL/min/1.73m^2^) could have led to a healthier reference group with lower comorbidities not reflecting the general elderly population.

In conclusion, this large nationwide study of patients with advanced CKD found that the 1-year risk of cardiovascular mortality was 9.8% for patients with diabetes and 7.4% for patients without diabetes. Diabetes remained an important risk factor of cardiovascular mortality across the range of advanced CKD stages and age groups, particularly in younger patients. Albuminuria and anemia were associated with increased risk of cardiovascular mortality regardless of diabetes status, whereas our data suggest a potential limitation of LDL-cholesterol as a predictor of cardiovascular mortality in advanced CKD. Increased awareness of cardiovascular risk assessment that considers the uremia-specific risk factors can help identify patients at high risk of death from cardiovascular causes and may improve the prognosis of this high-risk population.

## Electronic supplementary material

Below is the link to the electronic supplementary material.

Supplementary Material 1

## Data Availability

The datasets generated and analyzed during the current study are not publicly available due to the risk of potential re-identification of persons but are available from the corresponding author on reasonable request.

## Abbreviations

ATC: Anatomical Therapeutic Chemical Classification System
CI: Confidence interval
CKD: Chronic kidney disease
eGFR: Estimated glomerular filtration rate
HR: Hazard ratio
ICD-10: 10th edition of the International Classification of Diseases
IQR: Interquartile range
LDL: Low-density lipoprotein
NCSP: Nordic Medico-Statistical Committee Classification of Surgical Procedures
NPU: Nomenclature, Properties and Units
RR: Risk ratio
SD: Standard deviation
UACR: Urinary albumin-to-creatinine ratio
