# Supplementary material for: Cardiovascular mortality in patients with advanced chronic kidney disease with and without diabetes: a nationwide cohort study
Source: Cardiovasc Diabetol. 2023 Jun 16;22:140. doi: 10.1186/s12933-023-01867-8 (PMC10276454; doi:10.1186/s12933-023-01867-8)

**Supplementary material**

Supplementary Table S1. Applied administrative codes.

Supplementary Table S2. Hazard ratios and standardized 1-year risk of cardiovascular mortality stratified by sex and cardiovascular disease (CVD).

Supplementary Table S3. Hazard ratios of cardiovascular mortality stratified by albuminuria, plasma low-density lipoprotein (LDL) cholesterol, and plasma hemoglobin level, respectively, and chronic kidney disease (CKD) stage.

Supplementary Table S4. Sensitivity analysis: Hazard ratios and standardized 1-year risk of cardiovascular mortality stratified by index year.

Supplementary Table S5. Sensitivity analysis: Hazard ratios and standardized 1-year risk of cardiovascular mortality in a population with two measured eGFR <30 mL/min/1.73m^2^ ≥90 days apart.

Supplementary Table S6. Sensitivity analysis: Hazard ratios and standardized 1-year risk of cardiovascular mortality in a population with two measured eGFR <30 mL/min/1.73m^2^ ≥90 days apart with a maximum of 20% variation between first and second eGFR.

Supplementary Figure S1. Cumulative incidence of cardiovascular, non-cardiovascular, and all-cause mortality.

Supplementary Figure S2. Standardized risk of non-cardiovascular and all-cause mortality.

Supplementary Figure S3. Standardized risk of cardiovascular mortality in patients without medication for hyperlipidemia.

**Supplementary Table S1. Applied administrative codes**

**International Classification of Diseases (ICD-10) codes pertaining to comorbidities**

| **Diagnosis** | **ICD-10 code** |
| --- | --- |
| Heart failure | DI50 |
| Myocardial infarction | DI21 |
| Atrial fibrillation/flutter | DI48 |
| Stroke | DI63, DI64 |
| Peripheral artery disease | DI739 |
| Diabetic eye disease | DH33, DH34, DH35, DH36, DH42, DH43, DE133, DE143, DE103, DE113 |

**Nordic Medico-Statistical Committee Classification (NCSP) billing codes pertaining to in-hospital procedures**

| **Procedure** | **NCSP code** |
| --- | --- |
| Kidney transplantation | KKAS |
| Chronic dialysis | BJFD2 |
| Lower extremity amputation | KNHQ1, KNFQ19, KNGQ19, KNFQ99, KNGQ99 |
| Diabetic eye disease | BCDE, BCHY8A, KCK |

**Anatomical Therapeutic Chemical Classification System (ATC) codes pertaining to prescription medication**

| **Prescription medication** | **ATC code** |
| --- | --- |
| Glucose-lowering medication | A10 |
| Antihypertensive medication incl. diuretics | C02-09 |
| Acetylsalicylic acid | B01AC06 |
| Lipid modifiers | C10 |

**Nomenclature, Properties and Units (NPU) codes pertaining to laboratory workup**

| **Laboratory workup** | **NPU code** |
| --- | --- |
| Albuminuria | NPU03277, NPU19661 |
| Hemoglobin | NPU02319 |
| Total cholesterol | NPU01566 |
| Low-density lipoprotein cholesterol | NPU01568 |
| High-density lipoprotein cholesterol | NPU01567 |
| Hemoglobin A1c | NPU273 |

**Supplementary Table S2. Hazard ratios and standardized 1-year risk of cardiovascular mortality stratified by sex and cardiovascular disease (CVD)**

|  | **Hazard ratio (95% CI)** | **Standardized  1-year risk  (95% CI)** | **Standardized risk  difference  (95% CI)** | **Standardized risk ratio  (95% CI)** | **P-value** |
| --- | --- | --- | --- | --- | --- |
| **Men** |  |  |  |  |  |
| Matched cohort | Reference | 3.2% (3.1-3.2) | Reference | Reference |  |
| No diabetes | 3.1 (3.0-3.1) | 8.6% (8.4-8.8) | 5.4% (5.3-5.6) | 2.7 (2.7-2.8) | <0.001 |
| Diabetes | 4.3 (4.1-4.4) | 11.6% (11.3-12.0) | 8.5% (8.1-8.8) | 3.7 (3.6-3.8) | <0.001 |
| **Women** |  |  |  |  |  |
| Matched cohort | Reference | 3.0% (3.0-3.0) | Reference | Reference |  |
| No diabetes | 2.4 (2.3-2.4) | 6.6% (6.4-6.7) | 3.5% (3.4-3.7) | 2.2 (2.1-2.2) | <0.001 |
| Diabetes | 3.0 (2.9-3.1) | 8.2% (7.9-8.5) | 5.2% (4.9-5.5) | 2.7 (2.6-2.8) | <0.001 |
| **CVD** | | | | | |
| Matched cohort | Reference | 7.7% (7.5-7.8) | Reference | Reference |  |
| No diabetes | 1.9 (1.9-2.0) | 13.7% (13.4-14.0) | 6.0% (5.8-6.3) | 1.8 (1.7-1.8) | <0.001 |
| Diabetes | 2.7 (2.6-2.8) | 18.2% (17.6-18.7) | 10.5% (10.0-11.1) | 2.4 (2.3-2.5) | <0.001 |
| **No CVD** |  |  |  |  |  |
| Matched cohort | Reference | 2.4% (2.4-2.5) | Reference | Reference |  |
| No diabetes | 3.1 (3.1-3.2) | 6.8% (6.7-6.9) | 4.4% (4.3-4.5) | 2.8 (2.8-2.9) | <0.001 |
| Diabetes | 4.3 (4.1-4.4) | 9.2% (8.9-9.5) | 6.8% (6.5-7.0) | 3.8 (3.7-3.9) | <0.001 |

**Supplementary Table S3. Hazard ratios of cardiovascular mortality stratified by albuminuria, low-density lipoprotein (LDL) cholesterol, and hemoglobin level, respectively, and chronic kidney disease (CKD) stage**

|  | **CKD stage 4** |  | **CKD stage 5** |  | **CKD stage 5D** |  |
| --- | --- | --- | --- | --- | --- | --- |
|  | **Hazard ratio (95% CI)** | **P-value** | **Hazard ratio (95% CI)** | **P-value** | **Hazard ratio (95% CI)** | **P-value** |
| **Albuminuria** |  |  |  |  |  |  |
| *Diabetes* |  |  |  |  |  |  |
| Normoalbuminuria | Reference |  | Reference |  | N/A |  |
| Microalbuminuria | 1.4 (1.2-1.5) | <0.001 | 1.4 (0.9-2.1) | 0.11 | N/A |  |
| Macroalbuminuria | 1.6 (1.4-1.8) | <0.001 | 1.9 (1.3-2.8) | 0.001 | N/A |  |
| *No diabetes* |  |  |  |  |  |  |
| Normoalbuminuria | Reference |  | Reference |  | N/A |  |
| Microalbuminuria | 1.3 (1.2-1.5) | <0.001 | 0.9 (0.6-1.4) | 0.75 | N/A |  |
| Macroalbuminuria | 1.7 (1.5-1.9) | <0.001 | 1.5 (1.0-2.0) | 0.03 | N/A |  |
| **LDL-cholesterol level** |  |  |  |  |  |  |
| *Diabetes* |  |  |  |  |  |  |
| <1.8 mmol/L | Reference |  | Reference |  | N/A |  |
| 1.8-2.6 mmol/L | 0.9 (0.8-1.0) | 0.003 | 0.9 (0.7-1.2) | 0.50 | N/A |  |
| >2.6 mmol/L | 1.0 (0.9-1.1) | 0.62 | 1.0 (0.7-1.4) | 1.00 | N/A |  |
| *No diabetes* |  |  |  |  |  |  |
| <1.8 mmol/L | Reference |  | Reference |  | N/A |  |
| 1.8-2.6 mmol/L | 0.9 (0.8-0.9) | <0.001 | 0.9 (0.7-1.1) | 0.21 | N/A |  |
| >2.6 mmol/L | 0.8 (0.7-0.8) | <0.001 | 0.8 (0.6-1.0) | 0.03 | N/A |  |
| **Hemoglobin level** |  |  |  |  |  |  |
| *Diabetes* |  |  |  |  |  |  |
| <6.5 mmol/L | Reference |  | Reference |  | Reference |  |
| 6.5-7.5 mmol/L | 0.8 (0.8-0.9) | <0.001 | 0.9 (0.7-1.0) | 0.11 | 0.9 (0.6-1.3) | 0.52 |
| >7.5 mmol/L | 0.7 (0.7-0.8) | <0.001 | 0.7 (0.5-0.8) | <0.001 | 0.6 (0.4-1.0) | 0.04 |
| *No diabetes* |  |  |  |  |  |  |
| <6.5 mmol/L | Reference |  | Reference |  | Reference |  |
| 6.5-7.5 mmol/L | 0.8 (0.8-0.9) | <0.001 | 0.8 (0.7-0.9) | 0.004 | 0.8 (0.6-1.1) | 0.10 |
| >7.5 mmol/L | 0.7 (0.7-0.8) | <0.001 | 0.7 (0.6-0.8) | <0.001 | 0.6 (0.4-0.8) | 0.002 |

N/A due to few observations.

**Supplementary Table S4. Sensitivity analysis: Hazard ratios and standardized 1-year risk of cardiovascular mortality stratified by index year**

|  | **Hazard ratio (95% CI)** | **Standardized  1-year risk  (95% CI)** | **Standardized risk  difference  (95% CI)** | **Standardized risk ratio  (95% CI)** | | **P-value** |
| --- | --- | --- | --- | --- | --- | --- |
| **Year 2002-2008 (n=165,320)** | | | | | | |
| Matched cohort | Reference | 3.7% (3.7-3.8) | Reference | Reference | |  |
| No diabetes | 2.5 (2.5-2.6) | 8.4% (8.2-8.6) | 4.7% (4.5-4.9) | 2.3 (2.2-2.3) | | <0.001 |
| Diabetes | 3.5 (3.3-3.6) | 11.2% (10.8-11.7) | 7.5% (7.1-7.9) | 3.0 (2.9-3.1) | | <0.001 |
| **Year 2009-2013 (n=160,985)** | | | | |  |  |
| Matched cohort | Reference | 3.2% (3.1-3.2) | Reference | Reference | |  |
| No diabetes | 2.6 (2.5-2.7) | 7.5% (7.3-7.6) | 4.3% (4.1-4.5) | 2.3 (2.3-2.4) | | <0.001 |
| Diabetes | 3.3 (3.2-3.5) | 9.4% (9.0-9.8) | 6.2% (5.8-6.7) | 3.0 (2.8-3.1) | | <0.001 |
| **Year 2014-2018 (n=366,600)** | | | | |  |  |
| Matched cohort | Reference | 2.7% (2.6-2.7) | Reference | Reference | |  |
| No diabetes | 3.2 (3.1-3.3) | 7.6% (7.4-7.8) | 4.9% (4.8-5.1) | 2.9 (2.8-3.0) | | <0.001 |
| Diabetes | 3.8 (3.7-4.0) | 9.0% (8.7-9.3) | 6.3% (6.0-6.6) | 3.4 (3.2-3.5) | | <0.001 |

**Supplementary Table S5. Sensitivity analysis:** **Hazard ratios and standardized 1-year risk of cardiovascular mortality in a population with two measured eGFR <30 mL/min/1.73m^2^ ≥90 days apart**

|  | **Hazard ratio (95% CI)** | **Standardized  1-year risk  (95% CI)** | **Standardized risk  difference  (95% CI)** | **Standardized risk ratio  (95% CI)** | **P-value** |
| --- | --- | --- | --- | --- | --- |
| Matched cohort (n=288,224) | Reference | 3.6% (3.6-3.7) | Reference | Reference |  |
| No diabetes (n=51,186) | 3.1 (3.0-3.1) | 9.9% (9.7-10.1) | 6.3% (6.1-6.5) | 2.7 (2.7-2.8) | <0.001 |
| Diabetes (n=20,872) | 4.1 (3.9-4.2) | 12.7% (12.4-13.0) | 9.1% (8.8-9.4) | 3.5 (3.4-3.6) | <0.001 |

**Supplementary Table S6. Sensitivity analysis: Hazard ratios and standardized 1-year risk of cardiovascular mortality** **in a population with two measured eGFR <30 mL/min/1.73m^2^ ≥90 days apart with a maximum of 20% variation between first and second eGFR**

|  | **Hazard ratio (95% CI)** | **Standardized  1-year risk  (95% CI)** | **Standardized risk  difference  (95% CI)** | **Standardized risk ratio  (95% CI)** | **P-value** |
| --- | --- | --- | --- | --- | --- |
| Matched cohort (n=245,732) | Reference | 3.6% (3.6-3.7) | Reference | Reference |  |
| No diabetes (n=43,245) | 3.0 (2.9-3.1) | 9.8% (9.6-10.0) | 6.1% (6.0-6.3) | 2.7 (2.6-2.7) | <0.001 |
| Diabetes (n=18,190) | 4.0 (3.9-4.1) | 12.6% (12.2-12.9) | 8.9% (8.6-9.2) | 3.5 (3.4-3.5) | <0.001 |

**Supplementary Figure S1. Cumulative incidence of cardiovascular, non-cardiovascular, and all-cause mortality**

Cumulative incidence with 95% CIs of cardiovascular (Panel A), non-cardiovascular (Panel B), and all-cause mortality (Panel C) in patients with advanced chronic kidney disease and the matched cohort

**A. Cardiovascular mortality**

**B. Non-cardiovascular mortality**


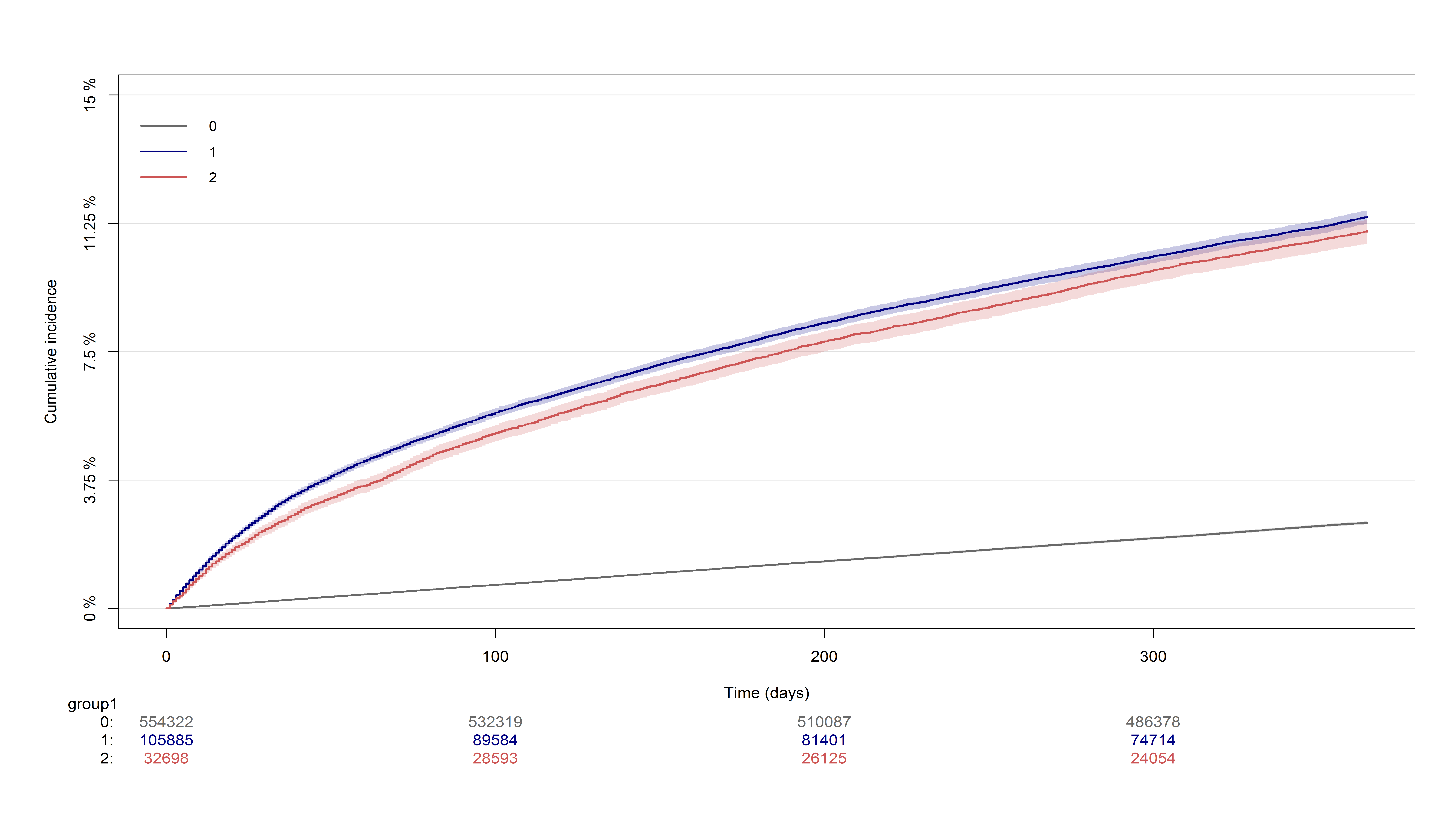

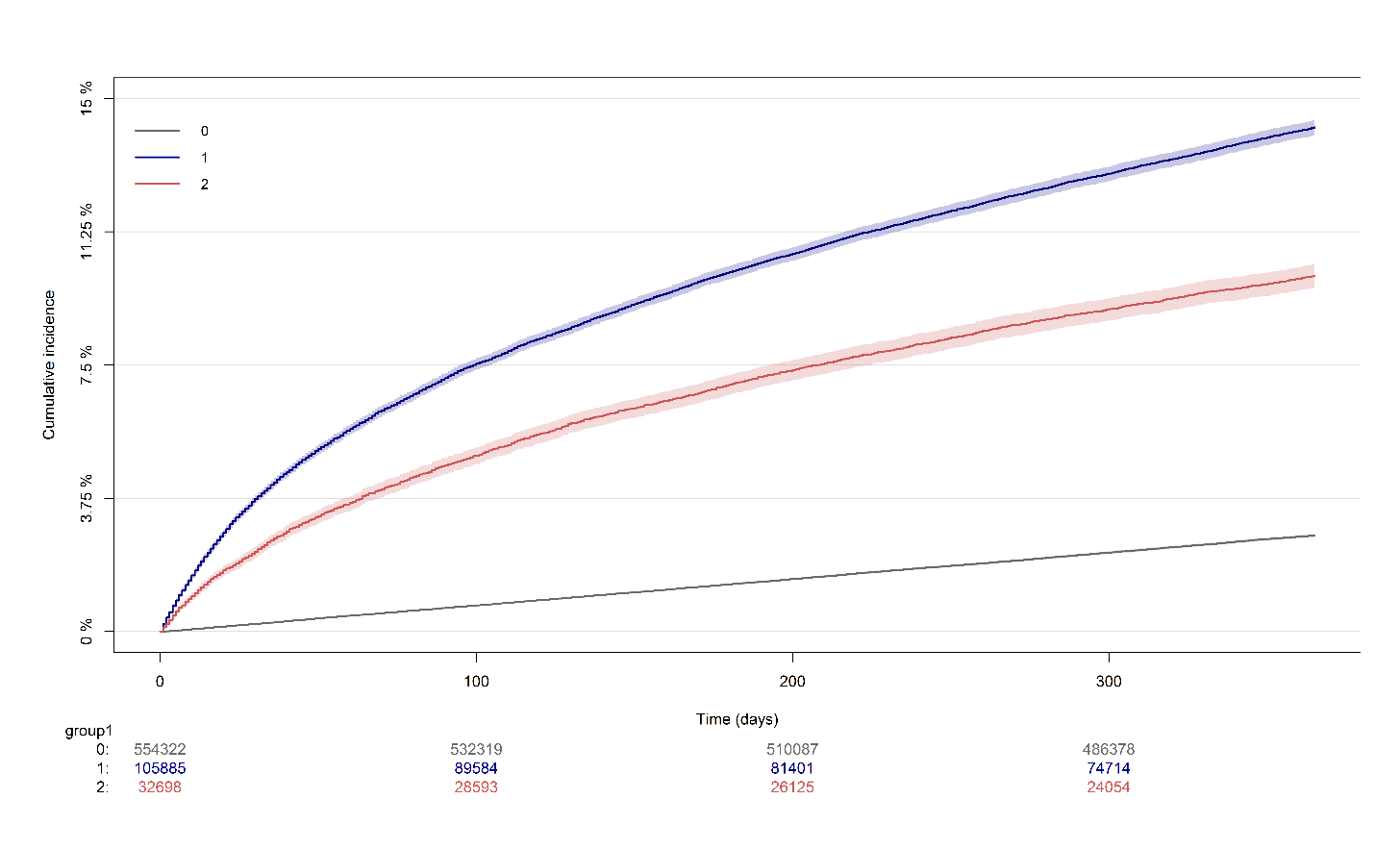

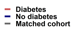

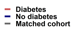

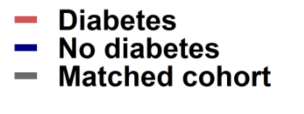

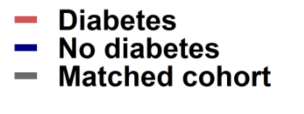


**C. All-cause mortality**


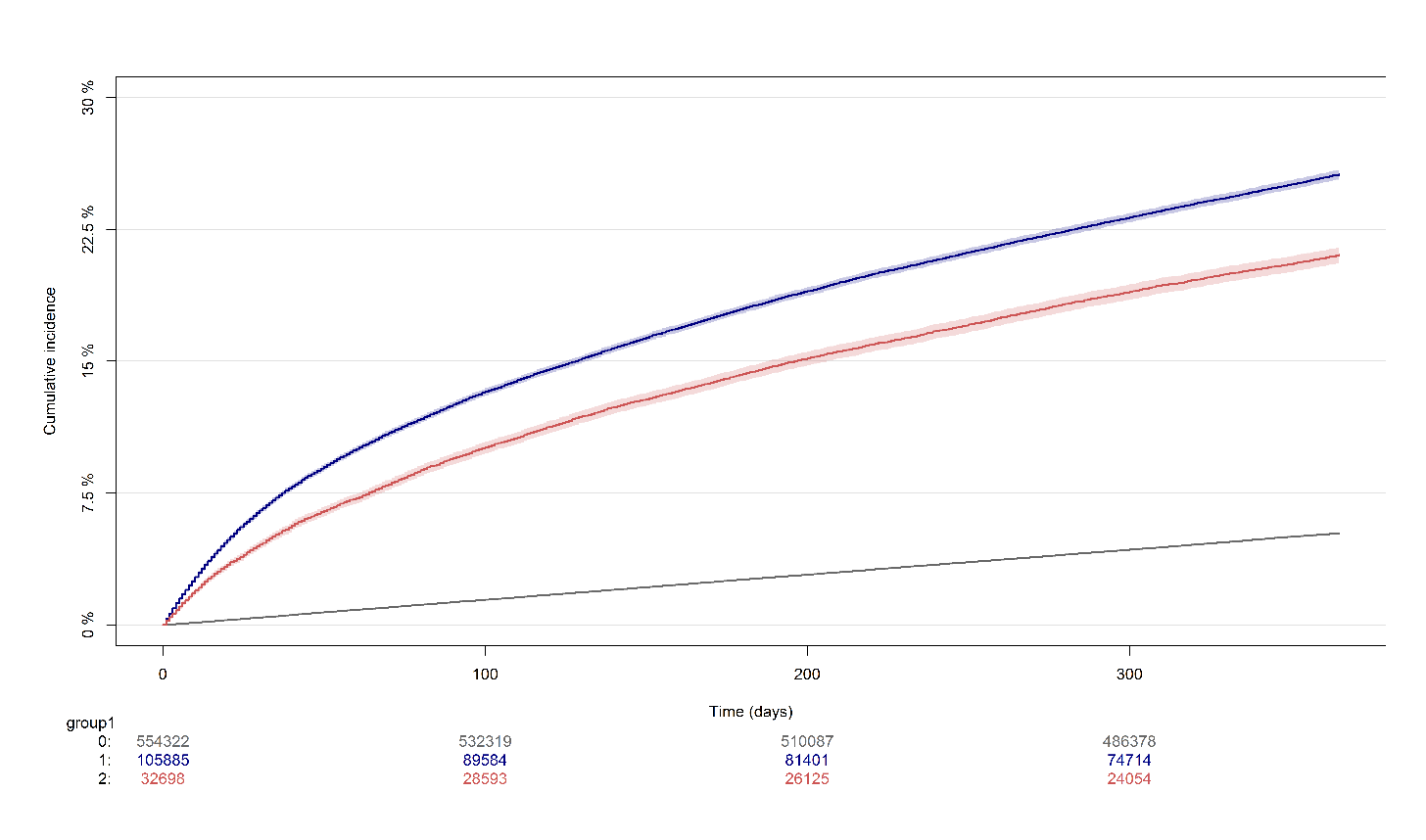

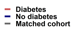

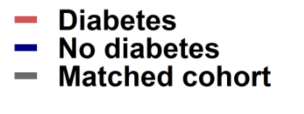


**Supplementary Figure S2. Standardized risk of non-cardiovascular and all-cause mortality**

Standardized risk with 95% CIs of non-cardiovascular (Panel A) and all-cause mortality (Panel B) in patients with advanced chronic kidney disease and the matched cohort

**A. Non-cardiovascular mortality**

**B. All-cause mortality**


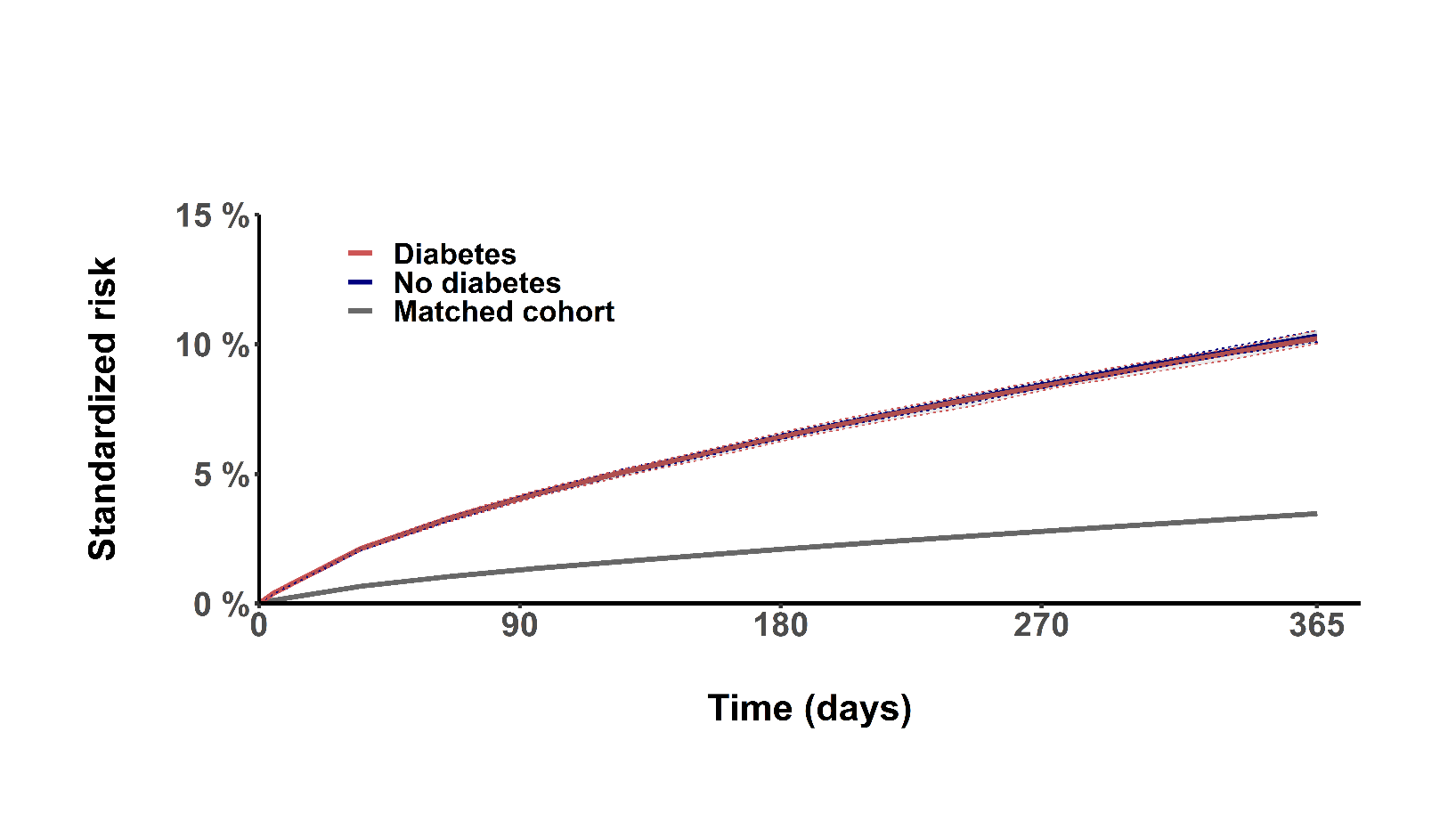

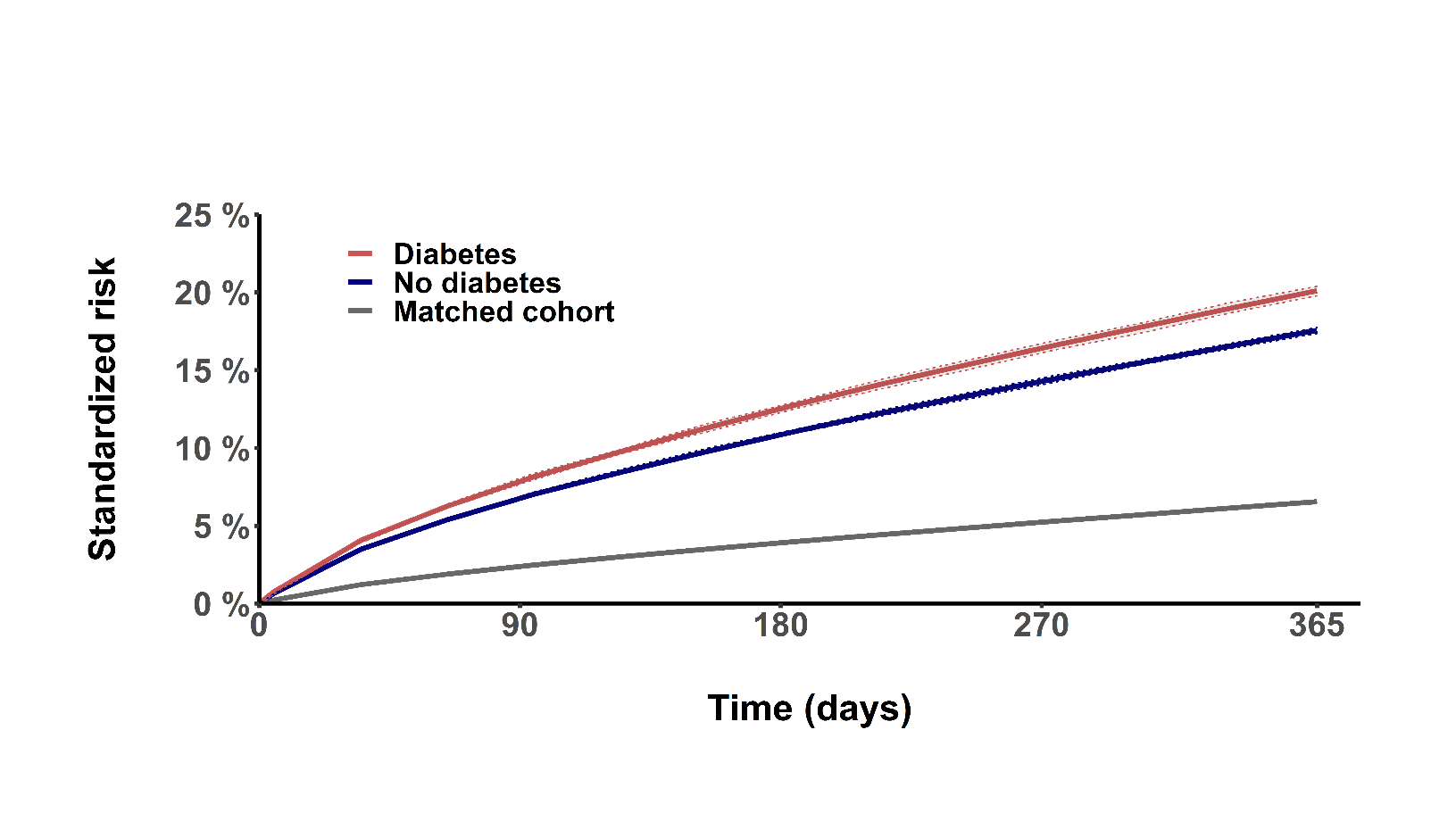


**Supplementary Figure S3. Standardized risk of cardiovascular mortality in patients without medication for hyperlipidemia**

Standardized risk with 95% CIs of cardiovascular mortality stratified by plasma low-density lipoprotein (LDL) cholesterol level in patients without concomitant medication for hyperlipidemia

**A. Diabetes (n=5,053)**

**B. No diabetes (n=20,859)**


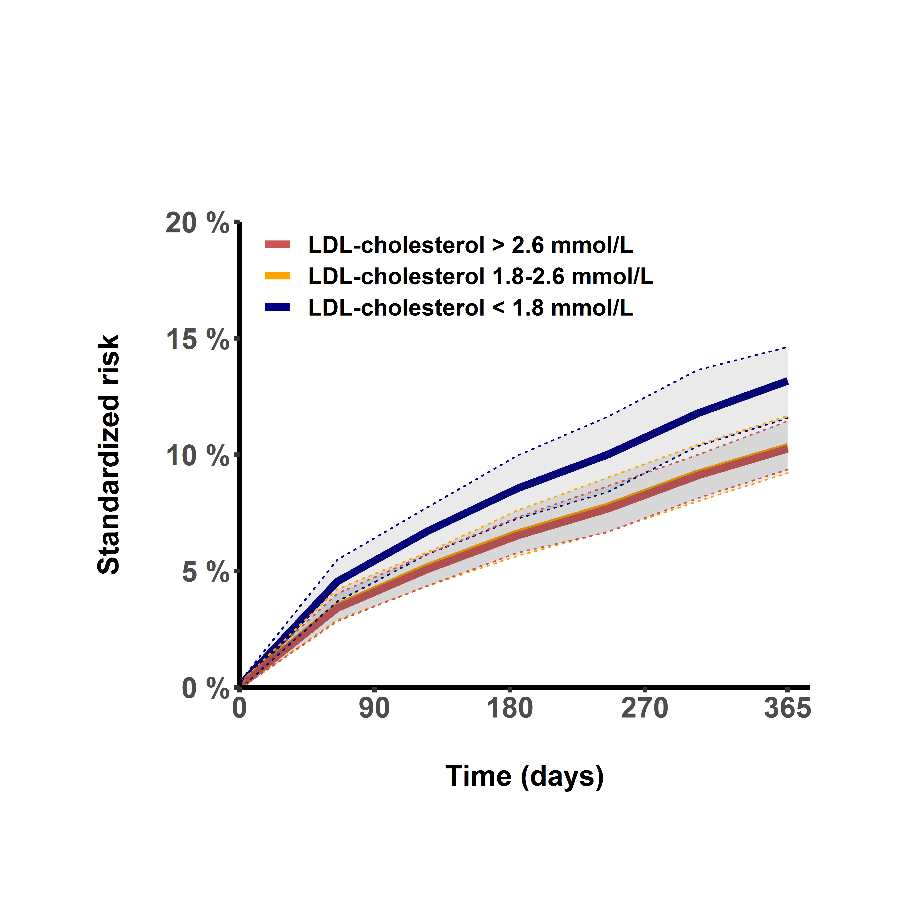

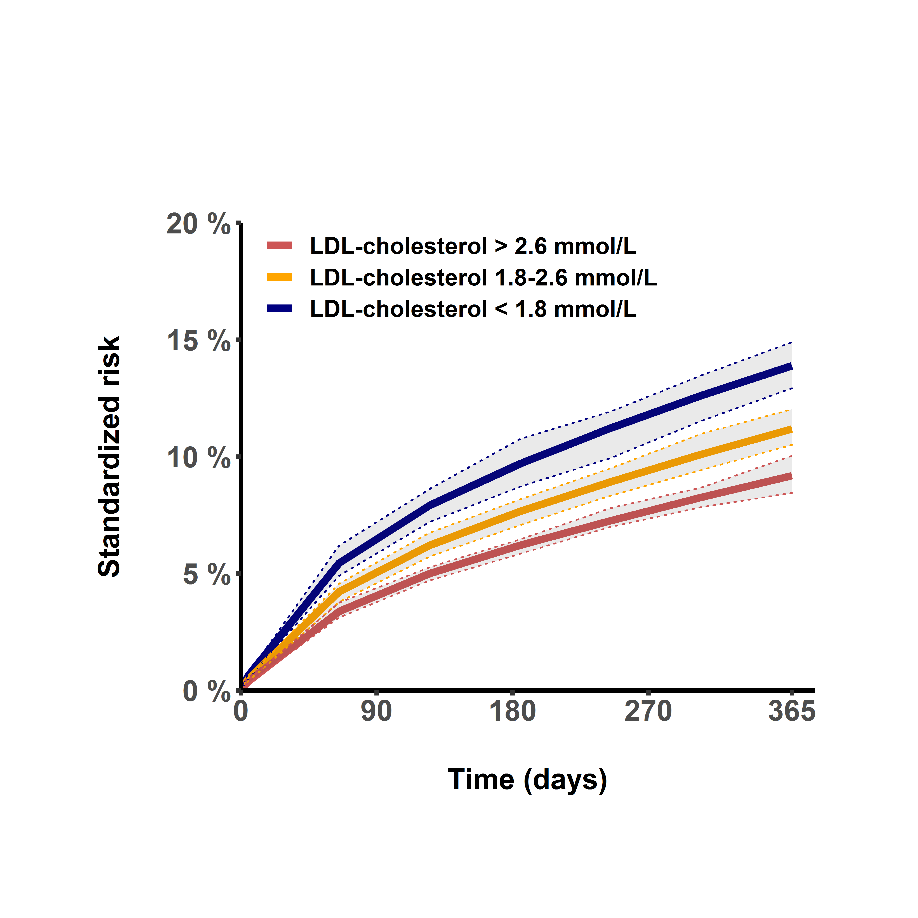

Supplement: Supplementary file 1 — Supplementary Material 1 [file 12933_2023_1867_MOESM1_ESM.docx]
